# Cyasterone ameliorates sepsis-related acute lung injury via AKT (Ser473)/GSK3β (Ser9)/Nrf2 pathway

**DOI:** 10.1186/s13020-023-00837-2

**Published:** 2023-10-19

**Authors:** Miao Lin, Weixi Xie, Dayan Xiong, Siyuan Tang, Xiaoting Huang, Lang Deng, Lei Huang, Xiaohua Zhang, Tingting Zhou, Rui Qian, Qian Zeng, Xiaoxue Sang, Yuyang Luo, Qingzhong Hua, Lu Ren, Wei Liu

**Affiliations:** 1 Department of Community Nursing, Xiangya Nursing School, Central South University, Changsha, 410013 China; 2 Occupational Disease Department, Hunan Prevention and Treatment Institute for Occupational Diseases, Changsha, 410013 China; 3 Clinical Nursing Teaching and Research Section, The Second Xiangya Hospital, Central South University, Changsha, 410013 China

**Keywords:** Cyasterone, Acute lung injury, Inflammation, Oxidative stress, Nrf2, AKT

## Abstract

**Background:**

Acute lung injury (ALI) is a severe disease that can lead to acute respiratory distress syndrome (ARDS), characterized by intractable hypoxemia, poor lung compliance, and respiratory failure, severely affecting patients' quality of life. The pathogenesis of ALI has not been fully elucidated yet, and sepsis is an important cause of ALI. Among the organ injuries caused by sepsis, the lungs are the earliest damaged ones. Radix cyathulae is reported to have analgesic, anti-inflammatory, and anti-aging effects. Cyasterone is extracted from Radix cyathulae. However, it is not known whether cyasterone has protective effects for ALI. This study aims to investigate the effect of cyasterone on sepsis-related ALI and its mechanism.

**Methods:**

We used the cecal ligation peferation (CLP) method to establish a mouse sepsis model, and cyasterone was given intraperitoneally on days 1–3 to observe its preventive effect on sepsis-related acute lung injury. Primary murine peritoneal macrophages were used to investigate the molecular mechanism of cyasterone in vitro.

**Results:**

Cyasterone pretreatment inhibits pro-inflammatory cytokine production, NLRP3 inflammasome activation, and oxidative stress in vivo and in vitro. In addition, cyasterone attenuates sepsis-induced ALI by activating nuclear factor erythroid2-related factor (Nrf2), which may be associated with AKT(Ser473)/GSK3β(Ser9) pathway activation.

**Conclusions:**

Cyasterone defends against sepsis-induced ALI by inhibiting inflammatory responses and oxidative stress, which depends heavily on the upregulation of the Nrf2 pathway through phosphorylation of AKT(Ser473)/GSK3β(Ser9). These results suggest cyasterone may be a valuable drug candidate for preventing sepsis-related ALI.

**Supplementary Information:**

The online version contains supplementary material available at 10.1186/s13020-023-00837-2.

## Introduction

Sepsis, defined by the World Health Organization (WHO) as a global health priority, is characterized by excessive inflammation after infection, with a mortality rate of 30–45% in hospitalized patients [1]. ALI/ARDS is the most common form of multi-organ dysfunction syndrome and an influential driver of sepsis morbidity and mortality [2]. Due to the lack of specific drugs, mechanical ventilation remains the primary supportive treatment for ALI/ARDS [3]. However, this may cause alveolar wall damage and further induce lung injury-related mechanical ventilation [4, 5]. Therefore, developing safe and effective drugs to reduce the mortality of sepsis patients by preventing the occurrence of ALI is significant.

As heterologous phagocytes in innate immunity, macrophages detect pathogen-associated molecular patterns (PAMP) and injury-associated molecular patterns by expressing pattern recognition receptors [6]. Infiltration of macrophages and neutrophils activated by inflammatory response into the lung activates pro-inflammatory networks and promotes reactive oxygen species (ROS) release [7]. In response to increasing ROS, thioredoxin-interacting protein (TXNIP) detaches from thioredoxin (Trx), binds to nucleotide-binding structural domain-like receptor protein 3 (NLRP3), and then activates NLRP3 inflammasome [8]. The activation of NLRP3 inflammasome further leads to the maturation and release of pro-inflammatory cytokines, such as interleukin-1β (IL-1β), tumor necrosis factor-α (TNF-α) and induces oxidative stress [9].

Nrf2 plays a critical role in many inflammatory and oxidative stress-related diseases [10], and it regulates almost all Nrf2 antioxidant response element (ARE) signals transcriptionally [11]. Kelch-1ike ECH- associated protein 1 (Keap1) is a repressor protein that binds to Nrf2 and promotes its degradation via the ubiquitin–proteasome pathway, a major regulator pathway of Nrf2 [12]. In addition to keap1-dependent Nrf2 regulation, growing evidence suggests that the GSK-3β-mediated regulatory pathway independent of keap1 is a crucial route for Nrf2 activation and, thus, protection against multifunctional organ damage [13].

Radix cyathulae is a traditional herb that promotes blood circulation, strengthens bones and muscles, and is known for its analgesic, anti-inflammatory, and anti-aging benefits [14–16]. Cyasterone is extracted from Radix cyathulae [17, 18]. Considering the known anti-inflammatory effects of cyasterone [19, 20], and the limitations of the current treatment of sepsis-related ALI, we investigated the protective effect of cyasterone against CLP-induced ALI and its mechanism.

## Materials and methods

### Animals

C57BL/6 mice (male, 6–8 weeks) were purchased from the Animal Center of Central South University and housed under specific pathogen-free conditions. After a week of adaptive feeding, to observe the preventive effect of cyasterone, mice were divided into five groups randomly: control, CLP, CLP + L, CLP + M, and CLP + H group. Mice were injected intraperitoneally (i. p.) with cyasterone (1, 5, or 25 mg/kg/d) for 3 days. To verify the preventive effect of cyasterone, the mice were randomly divided into five groups: control, CLP, CLP + M, CLP + M + ML385, and CLP + M + LY294002. Mice were injected intraperitoneally (i. p.) ML385 (30 mg/kg/d, selleck, China) or LY294002 (5 mg/kg/d, selleck, China) for 3 h following administration intraperitoneally (i. p.) with cyasterone (5 mg/kg/d) for 3 days. To compare the drug effects of cyasterone and the positive drug dexamethasone, mice were divided into four groups randomly: control, CLP, CLP + M, and CLP + DEX. Mice were injected intraperitoneally (i. p.) with cyasterone (5 mg/kg/d) or dexamethasone (5 mg/kg/d) for 3 days [21, 22]. Cyasterone (Cat#: S9414, Selleck, China) was diluted with 0.9% saline, containing 40% PEG300 (Macklin, China), 5% dimethyl sulfoxide (DMSO) (Macklin, China), and 5% Tween80 (Macklin, China) (v/v/v). This study was conducted following the welfare and ethical principles of experimental animals. It was approved by the Laboratory Animal Welfare and Ethical Committee of Central South University (Approval No. CSU-2022-0095).

### CLP model

Mice were fasted for 12 h prior to surgery. After anesthesia, the skin was disinfected, and the cecum was exposed by making a 1 cm incision toward the middle of the abdomen of the mice. The cecum was ligated with 4–0 braided silk thread through the midpoint between the root and the end of the cecum. A 21-gauge needle was inserted into the ligated cecum, and a small drop of intestinal contents was extruded to induce infection. Finally, the cecum was reset, and the incision was closed [23]. For the control group, the abdomen was opened, and then the incision was closed. 24 h later, lung tissue or bronchoalveolar lavage fluid (BALF) was obtained for subsequent experiments.

### Histopathological evaluation

The right mid-lung of the mice was fixed, embedded in 4% paraformaldehyde neutral buffer overnight, cut into 4 µm sections, and stained with hematoxylin–eosin [24]. The severity of the injury was graded from 0 to 5: alveolar wall intact without thickening, no inflammatory infiltrate, no congestion, 0; alveolar wall thickening, slight inflammatory cell infiltration, 1; alveolar wall thickening, slight inflammatory cell infiltration, capillary dilation, 2; alveolar wall thickening significantly, inflammatory cell infiltration, interstitial congestion, 3; alveolar wall thickening, severe inflammatory cell infiltration, diffuse distribution, destruction of alveolar structure, necrosis and decompensation of bronchial mucosa epithelial cells, and solidification of lung tissue, 4; the alveolar wall thickening, severe inflammatory cell infiltration, diffuse distribution, destruction of alveolar structure, and solidification of lung tissue, 5. The inflammation score was measured independently by three pathologists blinded to the experiment.

### Lung wet to dry (W/D) ratio

The left lung was removed and weighed on a precision electronic scale (BSA224S-CW; sartorius, Germany), then placed in an oven and baked at 56 ℃ for 48 h until a constant weight was obtained as dry weight. The W/D ratio was calculated to evaluate the degree of pulmonary edema.

### M1 macrophage activation

The proportion of M1 macrophages in BALF was determined by flow cytometry. Cells were stained with PE-conjugated anti-mouse F4/80 (Cat# E-AB-F0995UD, Elabscience, China), APC-conjugated anti-mouse CD80 (Cat# E-AB-F0992E, Elabscience, China), and FITC-conjugated anti-mouse CD11b (Cat# E-AB-F1081C, Elabscience, China). Briefly, F4/80 is employed for labeling macrophages in BALF, while CD80 is utilized for marking M1-type macrophages.

### Enzyme-linked immunosorbent assay (ELISA)

BALF was captured by 2 intratracheal injections of 0.8 ml of cooled phosphate buffered saline (PBS). BALF was centrifuged at 4 ℃ for 10 min at 1500 r/min, lysed in ACK lysis buffer for 5 min, washed twice with ice-cold PBS, and centrifuged for 5 min. Subsequently, the contents of tumor necrosis factor-alpha (TNF-α) and in-terleukin-1 beta (IL-1β) in BALF were measured using ELISA kits (Cat# TNF-α: 88–7324; IL-1β: 88–7013; Invitrogen, USA). The contents were assayed by comparison of the optical density (450 nm) with the standard curve [25].

### Measurement of MPO, MDA, GSH and SOD levels

Lung tissues were lysed in extraction buffer, and all procedures were conducted strictly according to the instructions of superoxide dismutase (SOD), myeloperoxidase (MPO), glutathione (GSH) and malondialdehyde (MDA) assay kits (Cat# SOD: A001-3-2; MPO: A044-1-1; GSH: A006-2-1; MDA: A003-1-2; Nanjing Jiancheng Bioengineering Institute, Nanjing, China).

### Cells culture

Primary murine peritoneal macrophages were isolated from C57BL/6 mice. Mice were injected with 3 ml of 3% thioglycolate (Sigma-Aldrich, St. Louis, MO, USA). 4 days later, peritoneal macrophages were obtained by intraperitoneal lavage with prechilled RPMI 1640 (Procell, Wuhan, China). Cells were collected and centrifuged at 1500 rpm for 10 min at 4 ℃, and the precipitate was resuspended in RPMI 1640. Cells were placed in 6-well plates (2 × 10^6^ cells/well) for protein detection and ROS assessment, 12-well plates (1 × 10^6^ cells/well) for RNA detection, and 24-well plates (0.5 × 10^6^ cells/well) for immunofluorescence staining. After 2 h, RPMI 1640 containing 10% neonatal bovine serum (NBS, Sigma, USA) and 1% streptomycin/penicillin (Gibco, Waltham, MA, USA) were replaced, and non-adherent cells were removed. Cells were cultured in a humified CO2 incubator at 37 ℃. MLE-12 cells (provided by the State Key Laboratory of Genetics, Changsha) were cultured with 100 U/ml of streptomycin/penicillin and fetal bovine serum (10%) in DMEM (Gibco, USA) and arranged in an incubator containing 5% CO_2_ with a suitable temperature (37 ℃).

All cells were incubated with cyasterone (10 μM, 30 μM or 100 μM) for 24 h before being stimulated with or without LPS (100 ng/ml, from Escherichia coli O111: B4, Sigma-Aldrich, USA) according to different requirements.

### CCK-8 assay

Cell viability was assayed with Cell Counting Kit-8 (CCK-8) (Dojindo, Kumamoto, Japan). Primary murine peritoneal macrophages and MLE12 cells were incubated on 96-well plates and treated with different concentrations of cyasterone (1 μM, 3 μM, 10 μM, 30 μM, 100 μM, 200 μM). After 24 h, 10 μl CCK-8 reagent was added to each well and incubated at 37 ℃ for 1 h. OD values were measured by 450 nm enzyme assay.

### Measurement of MDA, GSH and SOD levels

Primary peritoneal macrophages were lysed in extraction buffer, and all procedures were conducted strictly according to the instructions of superoxide dismutase (SOD), myeloperoxidase (MPO), glutathione (GSH) and malondialdehyde (MDA) assay kits (Cat# SOD: A001-3-2; MPO: A044-1-1; GSH: A006-2-1; MDA: A003-1-2; Nanjing Jiancheng Bioengineering Institute, Nanjing, China).

### Measurement of ROS content

Primary peritoneal macrophages and MLE12 cells were incubated with 20 μM 2′,7′-Dichlorodihydrofluorescein diacetate (DCFH-DA) (Thermo, D399) for 30 min at 37 ℃ in the dark and washed three times with pre-cooled PBS. Fluorescence microscopy (Nikon Ti-s, Tokyo, Japan) and flow cytometry (BD LSRFortessa, Franklin Lakes, NJ, USA) were performed to observe the production of ROS.

### The apoptosis assay

Apoptosis assays were performed using the Annexin V-FITC Apoptosis Detection Kit (Cat# C1062L, Beyotime, China) according to the manufacturer's instructions. Briefly, MLE12 cells were washed twice with cold PBS and resuspended in PBS at a concentration of 1 × 10^6^ cells/ml. Subsequently, cells were stained with 5 μl of FITC Annexin V and 5 μl of propidium iodide, and incubated at room temperature in the dark for 15 min. Finally, the samples were analyzed using flow cytometry (BD LSRFortessa, Franklin Lakes, NJ, USA), and the results were analyzed using Flowjo10 software.

### Real-time quantitative polymerase chain reaction (Q-PCR)

Total RNA from lung tissues and cells was extracted with TRIzol (Thermo Fisher Scientific, USA) and reverse transcribed into cDNA using a reverse transcription kit (Thermo Fisher Scientific, USA) in accordance with the manufacturer’s protocol. Q-PCR was performed using SYBR GREEN (Promega, USA) and Bio-Rad CFX96 Touch Real-Time PCR Detection System (Bio-Rad, USA) Q-PCR. PCR conditions were as follows. 95 ℃ for 2 min, followed by 40 cycles of 95 ℃ for 3 s and 60 ℃ for 30 s, plus a 60–95 ℃ melting curve. Data were expressed in C.T. values normalized to β-actin, and the fold change between control and treated groups was determined using the 2-ΔΔCt method. The sequences are shown in Table 1.

**Table 1:** Primer sequences for qPCR

Gene	Forward primer	Reverse primer
β-actin	GGCTGTATTCCCCTCCAT	CCAGTTGGTAACAATGCCATGT
*HO-1*	*ACCGCCTTCCTGCTCAACATTG*	*CTCTGACGAAGTGACGCCATCTG*
*NQO-1*	*GCGAGAAGAGCCCTGATTGTACTG*	*AGCCTCTACAGCAGCCTCCTTC*
*NLRP3*	*TACGGCCGTCTACGTCTTCT*	*CGCAGATCACACTCCTCAAA*
*ASC*	*GACAGTACCAGGCAGTTCGT*	*AGTCCTTGCAGGTCAGGTTC*
*Pro-caspase-1*	*CACAGCTCTGGAGATGGTGA*	*CTTTCAAGCTTGGGCACTTC*
*IL-6*	*CTTCTTGGGACTGATGCTGGTGAC*	*AGGTCTGTTGGGAGTGGTATCCTC*
*IL-1β*	*TCGCAGCAGCACATCAACAAGAG*	*AGGTCCACGGGAAAGACACAGG*
*TNF-α*	*GCCTCTTCTCATTCCTGCTTGTGG*	*GTGGTTTGTGAGTGTGAGGGTCTG*

### Western blot analysis

Lung tissue and cells were plunged in ice-cold RIPA lysis buffer with protease and phosphatase inhibitors (Abcam, Cambridge, UK). Per the manufacturer's instructions, protein concentrations were quantified using the BCA Protein Assay Kit (Beyotime Biotech, Shanghai, China). Briefly, protein samples were loaded and separated on SDS-PAGE gels, transferred to PVDF membranes (Bio-Rad, USA), and blocked with 5% (w/v) skimmed milk containing 0.1% PBS buffer Tween 20 (v/v) or 5% (w/v) protease free bovine serum Albumin (BSA) (Sigma-Aldrich, USA). Then the membranes were incubated with β-actin antibody (1:5000, Cat#: 81,115-1-RR, SAB), NLRP3 anti-body (1:2000, Cat#: 15,101, CST), pro-caspase-1/p10/p20 antibody (1:1000, Cat#: ab179515, Abcam),Heme Oxygenase 1 antibody (1:1000, Cat#: ab52947, Abcam), NQO1 antibody (1:3000, Cat#: ab80588, Abcam),p-GSK3β (S9) antibody (1:1000, Cat#: WL03518, WanleiBio), GSK3β antibody (1:300, Cat#: WL01456, WanleiBio), p-AKT (Ser473) antibody (1:500, Cat#: WLP001a, WanleiBio), AKT antibody (1:500, Cat#: WL0003b, WanleiBio)overnight at 4 ℃, and secondary antibodies (1: 5000, SAB) were labeled with horseradish peroxidase for 2 h at room temperature. After washing with PBST, bands were detected with Luminata^™^ Crescendo chemiluminescence horseradish peroxidase substrate (Millipore, USA) and scanned using GeneGnome XRQ imager (Syngene, UK).

### Immunofluorescence

Primary peritoneal macrophages were fixed in 4% paraformaldehyde for 15 h, permeabilized with 0.5% (v/v) Triton X-100 for 20 min, blocked with goat serum (ZSGB Bio, China) for 30 min, and then incubated with Nrf2 antibody (1:100, Proteintech, China) overnight at 4 ℃. After washing with PBST, samples were incubated with fluorescent secondary antibody (1:100, Proteintech, China) in PBST for 1 h at room temperature, and DAPI was used to stain cell nuclei for 5 min and observed under the fluorescence microscope (Nikon Ti-s, Tokyo, Japan).

### Statistical analysis

All experiments were independently repeated three times. All data were presented as means ± standard deviations and analyzed using GraphPad Prism 9.0. A one-way analysis of variance and Student-Newman-Kersee (SNK) tests were used to compare the groups. P value < 0.05 was defined as a statistically significant difference.

## Results

### Cyasterone attenuates inflammation and oxidative stress in CLP-induced ALI mice

**Fig. 1:**
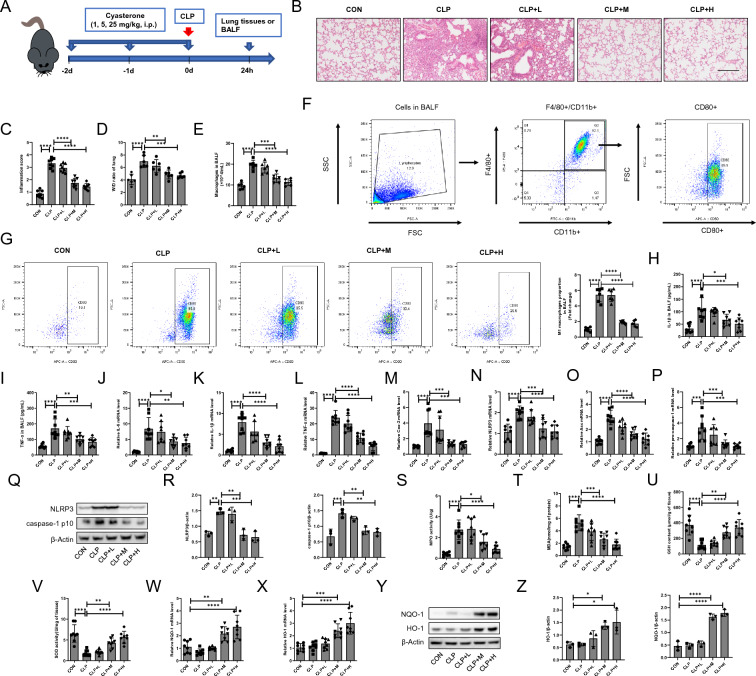
Cyasterone attenuates inflammation and oxidative stress in CLP-induced ALI mice. **A** Cyasterone was administered intraperitoneally at high (25 mg/kg), moderate (5 mg/kg), and low (1 mg/kg) doses to evaluate its prevention effects on CLP-induced ALI. **B**, **C** HE staining and lung inflammation score were used to detect the lung histopathological changes. Bars represent 100 μm. **D** Lung W/D ratio was measured to determine lung permeability. **E** The number of macrophages in BALF was measured. **F** The diagram of M1 Macrophage Cell Screening. **G** Flow cytometry was used to analyze and quantify the amount of CD80, an M1 macrophage marker in BALF. **H**, **I** IL-1β, TNF-α in BALF were determined with ELISA. **J-P** IL-6, IL-1β, TNF-α, Cox2, NLRP3, pro-caspase-1 and Asc mRNA in the lungs were determined with Q-PCR. **Q**, **R** Western blotting was used to detect the protein expression levels of NLRP3 and caspase-1 p10. **S**, **V** MPO activity, MDA content, GSH content and SOD activity in lung tissue were determined. **W–Z** Q-PCR and Western blotting were used to detect the mRNA and protein expression levels of NQO-1 and HO-1. Data are expressed as mean ± SD, n = 6–8, *P < 0.05; **P < 0.01; ***P < 0.001; ****P < 0.0001

To explore whether cyasterone can attenuate CLP-induced ALI in mice. The mice were intraperitoneally injected with different concentrations of cyasterone. CLP surgery was performed 1 h after cyasterone injection. 24 h later, the lungs and BALF were collected for subsequent experiments (Fig. 1A). HE staining was applied to appraise the pathological changes in lung tissues. The results showed alveolar wall thickening, interstitial inflammatory cell infiltration, and alveolar collapse in the CLP group mice (Fig. 1B, C). Notably, cyasterone attenuated these pathological changes (Fig. 1B, C) and reduced the lung wet/dry ratio (Fig. 1D). Macrophage count and flow cytometry results in BALF showed that macrophages were significantly elevated in the CLP group, and most of them were M1-type macrophages. In contrast, cyasterone pretreatment reduced the number of macrophages and inhibited M1-type macrophage activation (Fig. 1E–G). Next, we evaluated the effect of cyasterone on the degree of inflammation in ALI mice. The results showed that cyasterone pretreatment significantly reduced the expression of inflammatory factors in lung tissues and BALF (Fig. 1H, I, J-M) and inhibited the activation of NLRP3 inflammasome in lung tissues (Fig. 1N–R). Oxidative stress is one of the characteristics of ALI [26]. Cyasterone pre-treatment also inhibited the MPO activity, decreased the MDA content, increased the GSH content and restored the antioxidant enzyme SOD activity in lung tissues (Fig. 1S-V). In addition, our results showed that cyasterone pre-treatment promoted the expression of NQO-1 and HO-1 (Fig. 1W–Z). These results suggest that cyasterone attenuates the inflammation and increases antioxidant capacity in CLP-induced ALI mice. Further, the effects of cyasterone and dexamethasone in the prevention of ALI were compared, the results demonstrate that cyasterone and dexamethasone are capable of alleviating CLP-induced ALI. The differences in their effects do not hold statistical significance (Additional file 1: Fig. S1).

### Cyasterone inhibits the activation of NLRP3 inflammasome in primary murine macrophages

**Fig. 2:**
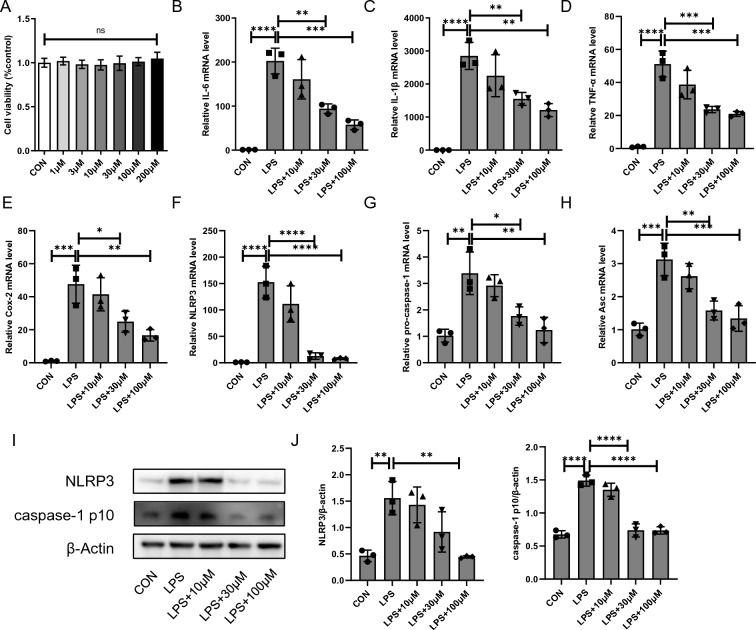
Cyasterone inhibits the activation of NLRP3 inflammasome in primary murine macrophages. **A** The effects of the different concentrations of cyasterone (0, 1, 3, 10, 30, 100 and 200 μM) on cell viability were evaluated. **B–J** Primary murine macrophages were treated with serial concentrations of cyasterone (10, 30 and 100 μM) for 24 h with or without LPS (100 ng/ml) for 12 h. **B–H** IL-6, IL-1β, TNF-α, Cox2, NLRP3, pro-caspase-1 and Asc mRNA were determined with Q-PCR. **I**, **J** Western blotting was used to detect the protein expression levels of NLRP3 and caspase-1 p10. Data are expressed as mean ± SD, n = 3, *P < 0.05; **P < 0.01; ***P < 0.001; ****P < 0.0001

To investigate the effect of cyasterone on LPS-induced NLRP3 activation in primary murine macrophages. Cells were treated with different doses of cyasterone (0, 1, 3, 10, 30, 100 and 200 μM) for 24 h. Results showed that cyasterone (1, 3, 10, 30, 100 and 200 μM) was not toxic to cells (Fig. 2A). We proceeded to appraise the efficacy of cyasterone on the inflammatory response of primary mouse macrophages and found that low doses of cyasterone (10 μM) had no significant effect on the expression of pro-inflammatory factors (Fig. 2B–E). However, cyasterone (30, 100 μM) pretreatment significantly reduced LPS-induced inflammatory factor expression, including IL-6, IL-1β, TNF-α, and COX-2 (Fig. 2B–E). Importantly, cyasterone pretreatment inhibited NLRP3 inflammasome activation (Fig. 2F–J). These results imply that cyasterone inhibits the activation of NLRP3 inflammasome in vitro.

### Cyasterone reduces oxidative stress in primary murine macrophages

**Fig. 3:**
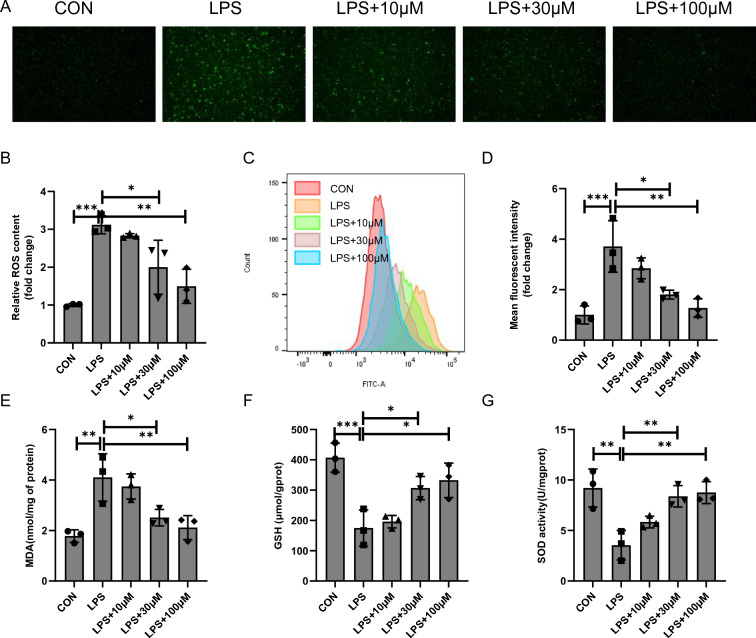
Cyasterone reduces oxidative stress in primary murine macrophages. Primary murine macrophages were treated with serial concentrations of cyasterone (10, 30 and 100 μM) for 24 h with or without LPS (100 ng/ml) for 12 h. **A**, **B** The ROS content was analyzed by fluorescence microscopy. **C**, **D** The ROS content was measured by flow cytometry. **E–G** MDA content, GSH content and SOD activity in macrophages were determined. Data are expressed as mean ± SD, n = 3, *P < 0.05; **P < 0.01; ***P < 0.001; ****P < 0.0001

ROS is among the most critical factors regulating NLRP3 inflammasome activation [9]. After LPS stimulation of cells, we measured intracellular ROS content. Both immunofluorescence and flow cytometric results found that cyasterone facilitated the subsidence of ROS (Fig. 3A–D). Cyasterone also reduced MDA content, increased GSH content and restored SOD activity in macrophages (Fig. 3E–G). Together, these results demonstrate that cyasterone attenuates oxidative stress levels in vitro.

### Cyasterone exerts anti-inflammatory and oxidative stress effects by activating Nrf2 in primary murine macrophages

**Fig. 4:**
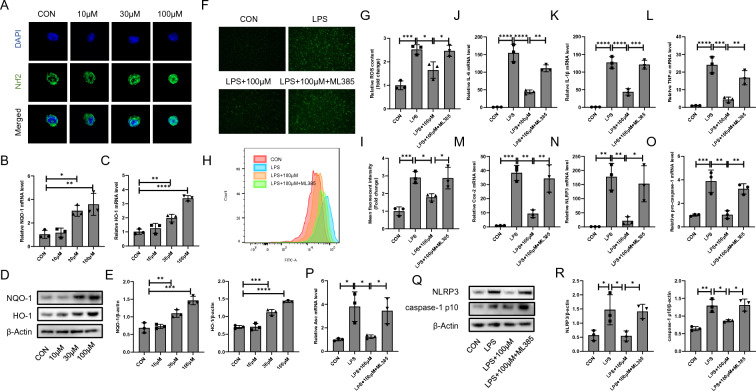
Cyasterone exerts anti-inflammatory and oxidative stress effects by activating Nrf2 in primary murine macrophages. Primary murine macrophages were treated with ML385 (3 μM) for 2 h following incubation with cyasterone (100 μM) for 24 h with or without LPS (100 ng/ml) for 12 h. **A** The expression of Nrf2 was analyzed by fluorescence microscopy. **B–E** Q-PCR and Western blotting were used to detect the mRNA and protein expression levels of NQO-1 and HO-1. **F-G** The ROS content was analyzed by fluorescence microscopy. **H-I** The ROS content was measured by flow cytometry. **J–P** IL-6, IL-1β, TNF-α, Cox2, NLRP3, pro-caspase-1 and Asc mRNA were determined with Q-PCR. **Q–R** Western blotting was used to detect the protein expression levels of NLRP3 and caspase-1 p10. Data are expressed as mean ± SD, n = 3, *P < 0.05; **P < 0.01; ***P < 0.001; ****P < 0.0001

Nrf2 transcriptionally regulates almost all antioxidant response element (ARE) pathways [11]. Therefore, we examined the effect of cyasterone on Nrf2 nuclear trans-location and showed that cyasterone facilitated Nrf2 entry into the nucleus concentration-dependently (Fig. 4A). It was shown that cyasterone could promote the mRNA and protein expression of Nrf2 downstream antioxidant enzymes NQO1 and HO-1 (Fig. 4B–E). After intervention with ML385 (Nrf2 inhibitor), we revealed that the role of cyasterone in inhibiting oxidative stress (Fig. 4F–I), reducing the expression of inflammatory factors (Fig. 4J–M), and suppressing NLRP3 inflammatory activation (Fig. 4N–R) were reversed to some extent. These results suggest that cyasterone inhibits NLRP3 inflammasome activation and promotes ROS clearance in macrophages by activating Nrf2.

### The AKT (Ser473)/GSK3β (Ser9) pathway mediates cyasterone-induced Nrf2 activation in primary murine macrophages

**Fig. 5:**
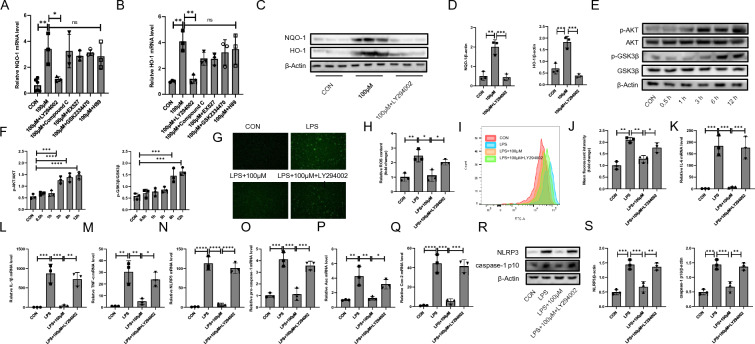
The AKT (Ser473)/GSK3β (Ser9) pathway mediates cyasterone induced Nrf2 activation in primary murine macrophages. Primary murine macrophages were treated with LY294002 (10 μM), Compound C (5 μM), EX527 (10 μM), GSK2334470 (1 μM), H89 (10 μM), respectively, for 2 h following incubation with cyasterone (100 μM) for 24 h with or without LPS (100 ng/ml) for 12 h. **A**, **B** NQO-1 and HO-1 mRNA were determined with Q-PCR. **C**, **D** Western blotting was used to detect the protein expression levels of NQO-1 and HO-1. **E**, **F** Primary murine macrophages were treated with cyasterone (100 μM) for 0, 0.5, 1, 3, 6,12 h. Western blotting was used to detect the protein expression levels of p-AKT, AKT, GSK3β and p-GSK3β. **G**, **H** The ROS content was analyzed by fluorescence microscopy. **I**, **J** The ROS content was measured by flow cytometry. **K-Q** IL-6, IL-1β, TNF-α, Cox2, NLRP3, pro-caspase-1 and Asc mRNA were determined with Q-PCR. **R**, **S** Western blotting was used to detect the protein expression levels of NLRP3 and caspase-1 p10. Data are expressed as mean ± SD, n = 3, *P < 0.05; **P < 0.01; ***P < 0.001; ****P < 0.0001

Various upstream signals can activate Nrf2 [27–29]. In this study, we explored the role of inhibitors of five essential kinases upstream of Nrf2, SIRT1, AMPK, AKT, PDK and PKA in the antioxidant and anti-inflammatory activities of cyasterone. Cells were pre-dressed by using the kinase inhibitors EX527, Compound C, LY294002, GSK2334470 and H89, respectively, and the results showed that LY294002 inhibits the mRNA levels of NQO-1 and HO-1 (Fig. 5A–D). Cyasterone could promote the phosphorylation of AKT Ser473 (activation) and GSK3β Ser9 (inactivation) in a time-dependent manner (Fig. 5E, F). The results further showed that both antioxidant and anti-inflammatory effects of cyasterone were inhibited after the inhibition of AKT (Fig. 5G–S). Taken together, the results suggest that cyasterone promotes Nrf2 into the nucleus via AKT (Ser473)/GSK3β (Ser9) pathway, thereby inhibiting inflammation and oxidative stress.

### Cyasterone against oxidative damage and apoptosis in MLE12 cells

**Fig. 6:**
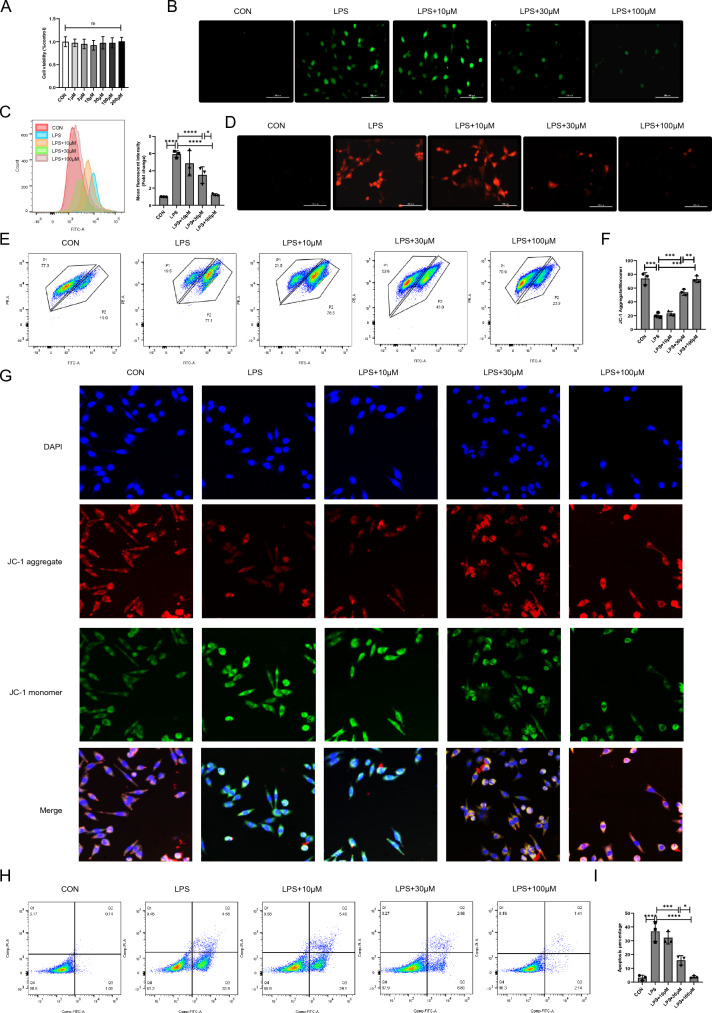
Cyasterone against oxidative damage and apoptosis in MLE12 cells. **A** The effects of the different concentrations of cyasterone (0, 1, 3, 10, 30, 100 and 200 μM) on cell viability were evaluated. **B–I** MLE12 cells were treated with serial concentrations of cyasterone (10, 30 or 100 μM) for 24 h with or without LPS (100 ng/ml) for 12 h. **B**, **C** The ROS content was analyzed by fluorescence microscopy and flow cytometry. **D** MitoSOX (5 μM) was measured by fluorescence microscopy. **E–G** Mitochondrial membrane potential (ΔΨm, MMP) was measured by flow cytometry and fluorescence microscopy with 5 μM JC-I. **H**, **I** The apoptosis of MLE12 cells was detected by flow cytometry. Data are expressed as mean ± SD, n = 3, *P < 0.05; **P < 0.01; ***P < 0.001; ****P < 0.0001

Given the role exerted by cyasterone in peritoneal macrophages, we proceeded to investigate the antioxidative capability of cyasterone in MLE12 cells. We initiated by assessing the impact of cyasterone (1, 3, 10, 30, 100 and 200 μM) on cell viability, and the CCK assay indicated that cyasterone did not inflict damage on MLE12 cells (Fig. 6A). Subsequently, after pre-treating the cells with cyasterone for 24 h followed by a 12h LPS stimulation, both immunofluorescence and flow cytometry results demonstrated that cyasterone (30, 100 μM) managed to reduce cellular and mitochondrial ROS levels (Fig. 6B–D). Notably, the JC-1 assay exhibited a concentration-dependent increase in the proportion of LPS-induced JC-1 aggregates in response to cyasterone (Fig. 6E–G). Furthermore, cyasterone effectively inhibited LPS-induced cell apoptosis (Fig. 6H, I). These results indicate that cyasterone could against oxidative damage and apoptosis in MLE12 cells.

### The AKT (Ser473)/ Nrf2 pathway mediates cyasterone against cell injury in MLE12 cells

**Fig. 7:**
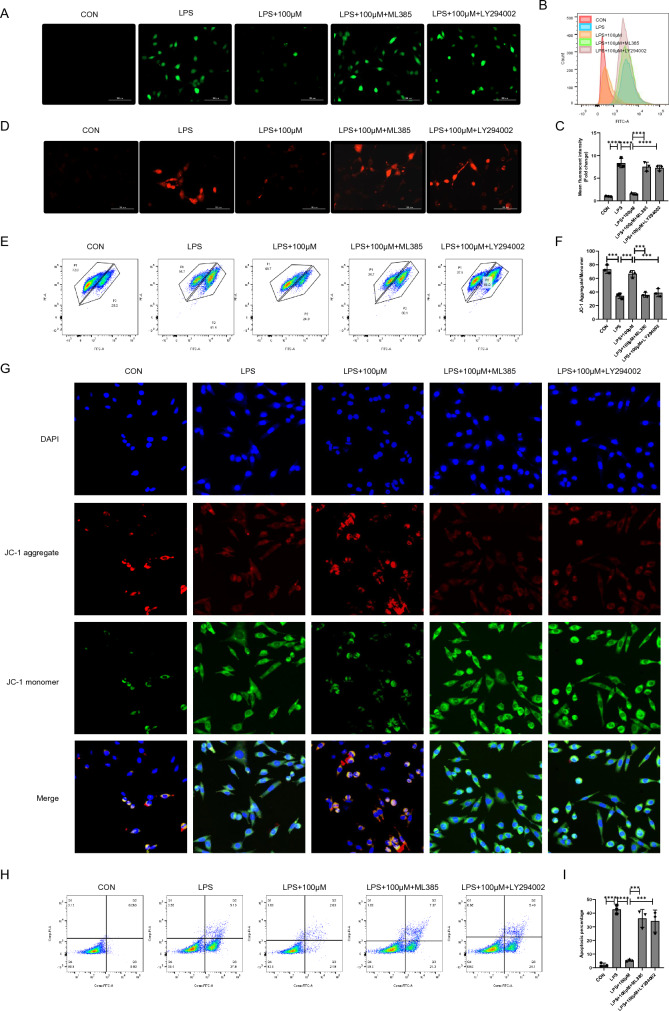
The AKT (Ser473)/ Nrf2 pathway mediates cyasterone against cell injury in MLE12 cells. **A**–**I** MLE12 cells were treated with ML385 (3 μM) or LY294002 (10 μM) for 2 h following incubation with cyasterone (100 μM) for 24 h with or without LPS (100 ng/ml) for 12 h. **A**–**C** The ROS content was analyzed by fluorescence microscopy and flow cytometry. **D** MitoSOX (5 μM) was measured by fluorescence microscopy. **E**–**G** Mitochondrial membrane potential (ΔΨm, MMP) was measured by flow cytometry and fluorescence microscopy with 5 μM JC-I. **H**, **I** The apoptosis of MLE12 cells was detected by flow cytometry. Data are expressed as mean ± SD, n = 3, *P < 0.05; **P < 0.01; ***P < 0.001; ****P < 0.0001

To deeper explore whether the inhibition role of cyasterone on oxidative damage in MLE12 cells through the AKT (Ser473)/Nrf2 pathway, we pre-treated MLE12 cells with AKT and Nrf2 inhibitors. The outcomes revealed that LY294002 and ML385 attenuated cyasterone’s capacity to alleviate cellular and mitochondrial ROS levels (Fig. 7A–D). Additionally, the JC-1 assay indicated that LY294002 and ML385 hindered the ability of cyasterone in increasing the proportion of JC-1 aggregates (Fig. 7E–G). Furthermore, the impact of cyasterone on inhibiting epithelial cell apoptosis was mitigated by LY294002 and ML385 (Fig. 7H, I).

### Cyasterone relieves the CLP-induced ALI via AKT (Ser473)/GSK3β (Ser9)/ Nrf2 pathway

**Fig. 8:**
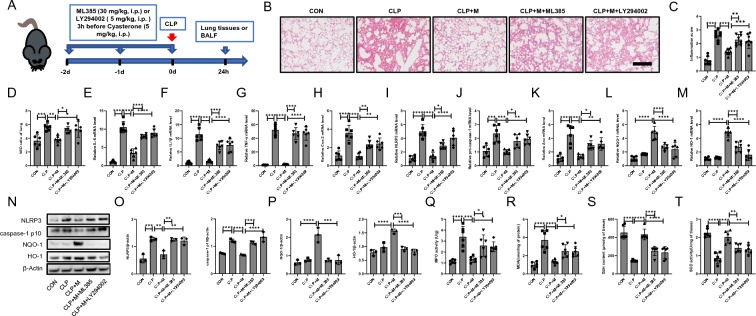
Cyasterone relieves the CLP-induced ALI by AKT (Ser473)/GSK3β (Ser9)/ Nrf2 pathway. **A** ML385 (30 mg/kg) or LY294002 (5 mg/kg) were injected intraperitoneally 3 h before the injection of cyasterone (5 mg/kg). **B**, **C** HE staining and lung inflammation score were used to detect the lung histopathological changes. Bars represent 100 μm. **D** Lung W/D ratio was measured to determine lung permeability. **E**–**M** IL-6, IL-1β, TNF-α, Cox2, NLRP3, pro-caspase-1, Asc, NQO-1 and HO-1 mRNA in the lungs were determined with Q-PCR. **N**–**P** Western blotting was used to detect the protein expression levels of NLRP3, caspase-1 p10, NQO-1 and HO-1. **Q**–**T** MPO activity, MDA content, GSH content and SOD activity in lung tissue were determined. Data are expressed as mean ± SD, n = 6–8, *P < 0.05; **P < 0.01; ***P < 0.001; ****P < 0.0001

To verify whether cyasterone attenuated CLP-induced acute lung injury in mice via the AKT (Ser473)/GSK3β (Ser9)/Nrf2 pathway, ML385 or LY294002 were injected intraperitoneally 3 h before the injection of cyasterone (Fig. 8A). HE staining results showed that ML385 and LY294002 groups of mice with disorganized lung Histological disorders (Fig. 8B, C). And ML385 and LY294002 reversed the effects of cyasterone in reducing the wet/dry ratio (Fig. 8D). In addition, the effect of cyasterone in decreasing the expression of inflammatory factors (Fig. 8E–H), inhibiting the activation of NLRP3 inflammasome (Fig. 8I–K, N–O), and anti-oxidative stress (Fig. 8L–N, P–T) were reversed by ML385 and LY294002. These results suggest that cyasterone alleviates the CLP-induced ALI via the AKT (Ser473)/GSK3β (Ser9)/ Nrf2 pathway (Figs. 8).

## Discussion

**Fig. 9:**
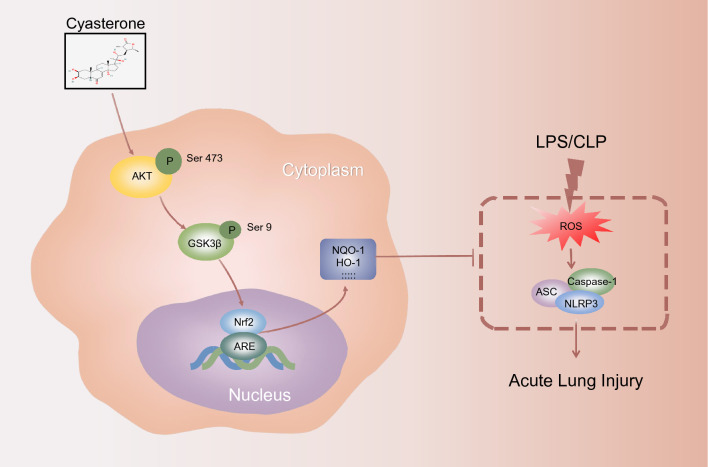
Schematic representation of the protective effect of cyasterone on CLP-induced ALI via AKT (Ser473)/GSK3β (Ser9)/Nrf2 axis. Cyasterone pretreatment activates AKT and GSK3β, promotes Nrf2 nucleation, increases the expression of antioxidant enzymes such as NQO-1 and HO-1, thereby reducing ROS content, inhibiting NLRP3 inflammasome activation in macrophages, and exerts a mitigating effect on CLP-induced ALI

Our study shows for the first time that cyasterone ameliorates sepsis-related ALI by inhibiting oxidative stress and inflammatory responses. We found that cyasterone pretreatment attenuated CLP-induced lung histopathological damage and oxidative stress, reduced inflammatory factor secretion, and inhibited NLRP3 inflammasome activation in ALI mice. Meanwhile, cyasterone increased the expression of antioxidant enzymes regulated by AKT(Ser473)/GSK3β(S9)/Nrf2 activation, decreased ROS content and thus reduced pro-inflammatory cytokine release and NLRP3 inflammasome activation stimulated by LPS in primary mouse peritoneal macrophages (Fig. 9). These results demonstrate that cyasterone may be a potential drug for preventing sepsis-related ALI.

Excessive inflammatory response plays a vital role in the development of ALI. Numerous studies have reported that NLRP3 inflammasomes function critically in ALI [30, 31]. LPS stimulation induces TXNIP binding to NLRP3, leading to NLRP3 inflammasome activation [32]. NLRP3 inflammasomes activation releases large amounts of mature IL-1β and IL-18, further amplifying the inflammatory response cascade [33]. Our results showed that cyasterone suppressed NLRP3 inflammasomes activation, suggesting that cyasterone protects against sepsis-associated ALI and may be related to inhibiting NLRP3 inflammasomes activation.

Oxidative stress is another essential characteristic of ALI [34]. Our study also showed that cyasterone attenuates oxidative stress in ALI mice and enhances antioxidant mechanisms involving Nrf2 activation. Extensive literature shows that ROS is one of the most vital factors regulating NLRP3 inflammasome activation [8, 9]. Although it was proposed in one study that Nrf2 is required for NLRP3 inflammasomes activation [35], another study showed that Nrf2 limits NLRP3 inflammasomes activation by downregulating ROS content [36]. Our results indicate that cyasterone can promote the expression of Nrf2 downstream antioxidant genes NQO-1 and HO-1 in a concentration-dependent manner, attenuating oxidative stress. Based on these results, we hypothesized that cyasterone could inhibit NLRP3 inflammasome activation by restoring the balance of oxidative and antioxidant mechanisms in ALI.

Normally, Nrf2 exists at low basal levels because the proteasome degrades it soon after synthesis [11]. It has been shown that Nrf2 activation can alleviate ALI in mice [37, 38]. Among them, Keap1, the fundamental mechanism of Nrf2 activation, is the main regulator of Nrf2 activity [39]. Under oxidative stress, the cysteine residues on Keap1 are oxidized, releasing Nrf2 from the Keap1/Nrf2 complex and subsequent translocation to the nucleus [40]. It has also been reported that GSK3β-mediated regulatory pathways independent of keap1 have a critical role in ALI induced by severe oxidative stress injury [13]. In addition to the ubiquitinated degradation of Nrf2 protein by phosphorylating specific serine residues of the Nrf2 Neh6 structural domain and forming a degradation domain recognized by the ubiquitin ligase adapter protein β-TrCP [41]. GSK3β can also export Nrf2 outside the cytoplasm by phosphorylating Fyn [42]. It is reported that sulforaphane can prevent chromium-induced lung injury in rats by activating the AKT/GSK-3β/Fyn pathway [43]. Another study also showed that melatonin prevented LPS-induced epithelial-mesenchymal transition in human alveolar epithelial cells via the GSK-3β/Nrf2 pathway [44]. Our results found that cyasterone can upregulate the expression of p-GSK-3β in a time gradient. Therefore, it is suggested that cyasterone can reduce Nrf2 degradation by inactivating GSK3β and cyasterone promotes Nrf2 entry into the nucleus, possibly by reducing GSK3β/β-TrCP-mediated Nrf2 degradation. However, further investigation needs to investigate whether cyasterone can reduce the nuclear export of Nrf2 by inhibiting Fyn kinase.

Multiple upstream signals can activate Nrf2 [27–29], and in this study, we selected inhibitors of SIRT1, AMPK, AKT, PDK and PKA, five important kinases upstream of Nrf2, and the results showed that inhibition of AKT can suppress the expression of NQO-1 and HO-1. PI3K/AKT is a multifunctional signaling pathway associated with cell proliferation, apoptosis and defense [45]. Mitochondrial quality control has been reported to have a significant role in septic lung oxidative injury through activation of PI3K/AKT pathway [46]. In contrast, LY294002, administered intraperitoneally, exacerbated the sepsis-related ALI [47]. Thus, drugs that enhance AKT activity may be novel agents for preventing sepsis-related lung injury. Our results also indicate that cyasterone can activate AKT time-dependently. Furthermore, the effect of cyasterone in reducing intracellular ROS content and inhibiting NLRP3 inflammasome activation was inhibited after the administration of LY294002.

Cyasterone is an active ingredient extracted from the traditional Chinese medicine Radix cyathulae [18]. It was demonstrated that in cancer cells A549 and MGC823, cyasterone blocked EGFR phosphorylation in a concentration-dependent manner, further inhibiting its downstream AKT phosphorylation [48]. However, cyasterone showed relatively high security in three normal human cells (HUVEC, L02, HEK293) [48]. Our results show that cyasterone activates AKT and inactivates GSK3β to promote its nuclear entry and antioxidant effects in mouse primary peritoneal macrophages.

## Conclusions

Our data reveal for the first time that cyasterone protects against CLP-induced ALI in mice. Cyasterone attenuates inflammation and oxidative stress in ALI mice, mainly attributed to the anti-inflammatory and antioxidant properties of cyasterone via AKT (Ser473)/GSK3β (Ser9)-mediated Nrf2 activation in macrophages and alveolar epithelial cell. These results suggest that cyasterone may be a potential drug candidate for preventing sepsis-induced ALI.

### Supplementary Information

**Additional file 1: Figure S1. **The effects of cyasterone and dexamethasone on alleviating CLP-induced ALI in mice showed no significant difference. **A **Cyasterone (5 mg/kg) or dexamethasone (5 mg/kg) was administered intraperitoneally to compare their effects on CLP-induced ALI. **B**, **C** HE staining and lung inflammation score were used to detect the lung histopathological changes. Bars represent 100 μm. **D** Lung W/D ratio was measured to determine lung permeability. **E** The number of macrophages in BALF was measured. **F** MPO activity in lung tissue were determined. **G**, **H** IL-1β, TNF-α in BALF were deter-mined with ELISA. **I-N** IL-6, IL-1β, TNF-α, NLRP3, pro-caspase-1 and Asc mRNA in the lungs were determined with Q-PCR. **O**, **P** Western blotting was used to detect the protein expression levels of NLRP3 and caspase-1 p10. Data are expressed as mean±SD, n = 6–8,*P<0.05;**P<0.01;***P<0.001; ****P<0.0001.

## Data Availability

All data generated or analyzed during this study are included in this published article.

## Abbreviations

ALI: Acute lung injury
ARDS: Acute respiratory distress syndrome
CLP: Cecal ligation peferation
LPS: Lipopolysaccharide
Nrf2: Nuclear factor erythroid2-related factor 2
AKT: Protein kinase A
GSK3β: Glycogen synthase kinase-3β
NLRP3: NOD-like receptor thermal protein domain associated protein 3
ROS: Reactive oxygen species
TXNIP: Thioredoxin-interacting protein
Trx: Thioredoxin
IL: Interleukin
TNF-α: Tumor necrosis factor-α
Cox-2: Cyclooxygenase-2
ARE: Antioxidant response element
BALF: Bronchoalveolar lavage fluid
