# Supplementary material for: Cyasterone ameliorates sepsis-related acute lung injury via AKT (Ser473)/GSK3β (Ser9)/Nrf2 pathway
Source: Chin Med. 2023 Oct 19;18:136. doi: 10.1186/s13020-023-00837-2 (PMC10585798; doi:10.1186/s13020-023-00837-2)
Supplement: Supplementary file 1 — Additional file 1: Figure S1. The effects of cyasterone and dexamethasone on alleviating CLP-induced ALI in mice showed no significant difference. A Cyasterone (5 mg/kg) or dexamethasone (5 mg/kg) was administered intraperitoneally to compare their effects on CLP-induced ALI. B, C HE staining and lung inflammation score were used to detect the lung histopathological changes. Bars represent 100 μm. D Lung W/D ratio was measured to determine lung permeability. E The number of macrophages in BALF was measured. F MPO activity in lung tissue were determined. G, H IL-1β, TNF-α in BALF were deter-mined with ELISA. I-N IL-6, IL-1β, TNF-α, NLRP3, pro-caspase-1 and Asc mRNA in the lungs were determined with Q-PCR. O, P Western blotting was used to detect the protein expression levels of NLRP3 and caspase-1 p10. Data are expressed as mean±SD, n = 6–8,*P<0.05;**P<0.01;***P<0.001; ****P<0.0001. [file 13020_2023_837_MOESM1_ESM.pdf]

Supplementary Figure 1

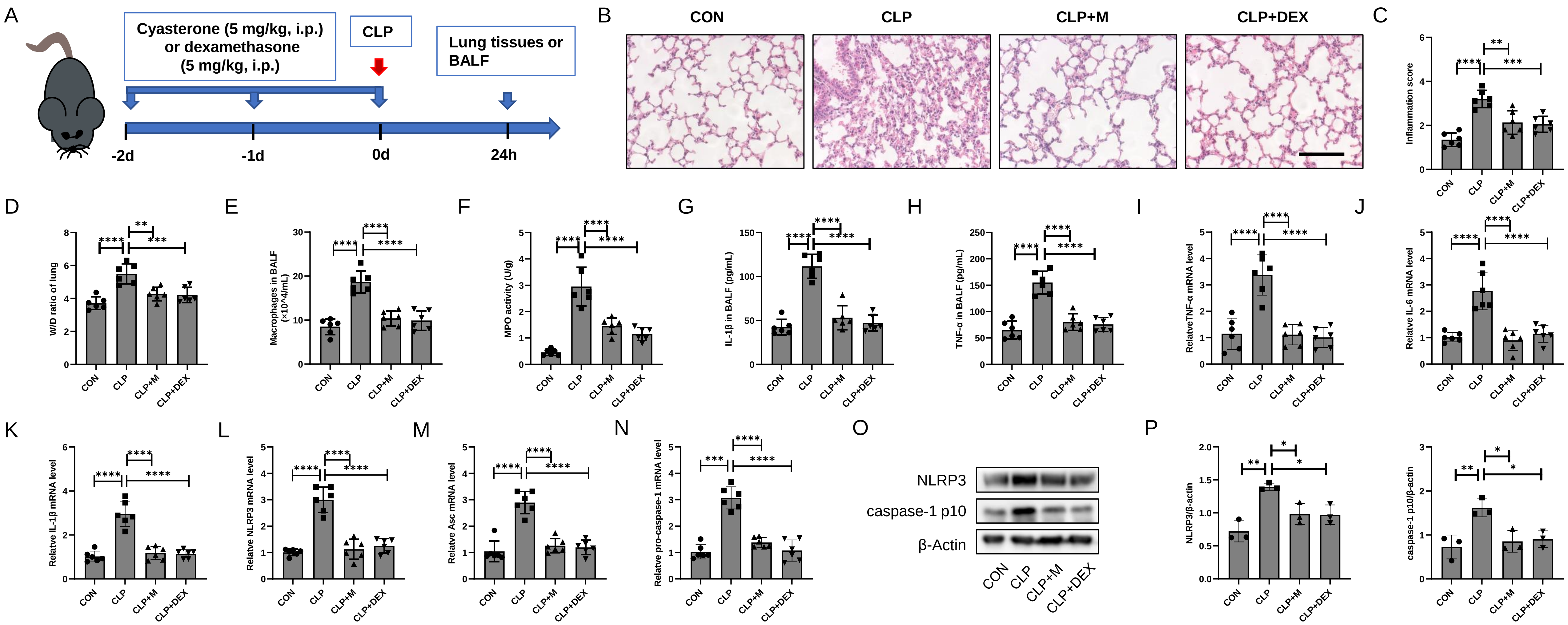

Supplementary Figure 1. Comparison of the effects of cyasterone and dexamethasone in CLP-induced ALI mice. (A) Cyasterone (5 mg/kg) or dexamethasone (5 mg/kg) was administered intraperitoneally to compare their effects on CLP-induced ALI. **(B-C)** HE staining and lung inflammation score were used to detect the lung histopathological changes. Bars represent 100 μm. **(D)** Lung W/D ratio was measured to determine lung permeability. **(E)** The number of macrophages in BALF was measured. **(F)** MPO activity in lung tissue were determined. **(G-H)** IL-1β, TNF-α in BALF were deter-mined with ELISA. **(I-N)** IL-6, IL-1β, TNF-α, NLRP3, pro-caspase-1 and Asc mRNA in the lungs were determined with Q-PCR. **(O-P)** Western blotting was used to detect the protein expression levels of NLRP3 and caspase-1 p10. Data are expressed as mean ± SD, n = 6–8, * $P < 0.05$; ** $P < 0.01$; *** $P < 0.001$; **** $P < 0.0001$.
